# Pulmonary Regurgitation- Is the Future Percutaneous or Surgical?

**DOI:** 10.3389/fped.2018.00184

**Published:** 2018-07-10

**Authors:** Gareth J. Morgan

**Affiliations:** Congenital Interventional Cardiologist, Heart Institute, Children's Hospital of Colorado, University Colorado Hospital, Colorado University, Denver, CO, United States

**Keywords:** percutaneous pulmonary valve, hybrid techniques, prosthetic valve endocarditis, minimally invasive surgery, Native outflow tract, pulmonary valve replacement

## Abstract

For decades, surgical replacement of the pulmonary valve has been seen as the gold-standard technique. Until the advent of Medtronic's Melody valve, it was the only option. Whilst radical changes in surgical techniques have not been forthcoming, rapid and substantial developments in the techniques and available technology for percutaneous valves now cause us to ask if the gold-standard moniker now belongs in the cath lab. This manuscript explores the recent history and future of a revolution in this large area of congenital cardiac practice.

## Introduction

Developments in percutaneous pulmonary valve technology are progressing rapidly. As such it can seem that transcatheter valve innovation is in a different league to surgical innovation. Can this statement be justified by an exploration of recent research in surgical techniques?

A review of the literature reveals the majority of surgical development is in the direction of innovative and collaborative hybrid techniques rather than traditional approaches (1, 2).

For the sake of our patients, we should try to avoid traditional surgical and interventional silos. Instead, by exploring techniques designed to minimize mortality, morbidity, and hospital stay we can tailor treatment to the patient's needs (3).

The last 10 years have seen steady progress in improvements in the technology and techniques around surgical valve replacement but precious little innovation in the core technology used. No company has aggressively developed a surgical pulmonary valve with improved longevity or performance; valve technology is still borrowed from aortic tissue valves which have, again reached a hard stop in their development. Stented tissue xenograft valves, cadaveric homografts and xenograft conduits remain the mainstay of treatment (4, 5). Operative techniques continue to be refined in general terms without any huge advances; in particular, cardiopulmonary bypass technology has not seen any major forward steps in over 10 years (6). We are aware of several ongoing studies, in particular those employing stem cell technology and tissue engineering techniques to mount tissue valves which should be resistant to the degradation which commits current valves to failure. Early results for these technologies are encouraging, and we must remember that there are no obvious barriers to the use of tissue engineering for percutaneously delivered valves in the future as well (7, 8). Unfortunately, precious little has been published in this area as industry research partnerships strive to achieve a practical and patentable product before revealing their techniques and methods in press.

Until we see a surgical product on the market which has truly innovative features, there is little impetus to lower the bar for surgical valve replacement nor to drive more patients from the percutaneous to the surgical method.

In comparison, there has been huge energy and impetus behind expanding the range of patients who can be treated with percutaneous techniques (9). This expansion can be tracked by looking from 2 different angles. The first is the development of innovative techniques to apply currently available technology, and the second is the development of new technology which is better suited to the patient need; overwhelmingly large dilated outflow tracts.

## Innovative techniques

### Hybrid techniques

Hybrid techniques have developed in response to two main pathophysiological conundrums. The first has been the desire for minimally invasive valve implantation in smaller patients in whom negotiating the right heart with large delivery systems produces too much haemodynamic instability. Overcoming this by directly cannulating the right ventricle allows positioning of the delivery sheath without interfering with the tricuspid valve or physical distorting the midcavity and outflow portion of the Right ventricle (10).

**Figure 1:**
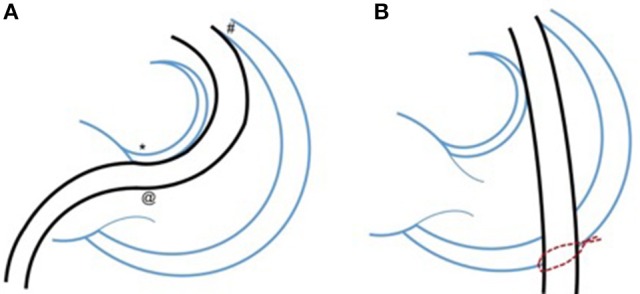
Minimizing distortion of the intracardiac anatomy with a hybrid approach. Demonstrating the potential benefit to smaller patients of a hybrid approach with lower partial “xiphi-sternotomy” which allows placement of a purse-string suture on the antero-inferior border of the right ventricle **(B)**. This in turn allows delivery of the relatively large valve delivery sheath, over a straight course across the right ventricular outflow tract and valve. **(A)** Shows the typical trans-femoral percutaneous route through the heart. The consistent issues with this approach are contact with the area of the AV node, which can lead to haemodynamically important arrhythmia; passage of the large sheath through the tricuspid valve, which may cause haemodynamic compromise and interaction with the anterior free wall of the outflow tract which can be difficult to navigate in post-operative patients and can also trigger important arrhythmias. Other interactions such as those with pacing wires, tricuspid surgical repairs, and calcified distorted outflow tracts can also cause problems.

By performing a limited lower sternotomy, splitting only the xiphisternum then placing a purse-string on the antero-inferior surface of the RV, the 22 F or 18 F Ensemble or Certitude systems for the Melody or Sapien valves respectively can be placed allowing fluoroscopic and angiographic guided valve placement (Figure 1). This technique still requires the regurgitant outflow tract to comply with the physical restraints which limit use of these valves when we deploy them percutaneously; mainly related to maximum diameter, but when used in smaller and smaller (hence younger and younger) patients, there has been less time for the outflow tract to progressively dilate and hence a higher proportion of outflows that should have suitable morphology and size.

The second category includes those patients in whom regurgitant outflows, due to years of persistent hyperdynamic activity secondary to PI have expanded beyond the range of currently available percutaneous valves. In order to decrease the morbidity of the operation (particularly in comorbid patients) the surgical component may be limited to the sternotomy and off-pump preparation of the outflow tract (11, 12). After opening the chest and dissecting down onto the pulmonary artery the surgeon can use one of many methods to decrease the circumference of the outflow. The method chosen by our team has been to suture to 5 cm long Teflon strips parallel and along the length of the outflow tract ~10–15 mm apart (depending on the amount of plication needed to accommodate the chosen valve.

**Figure 2:**
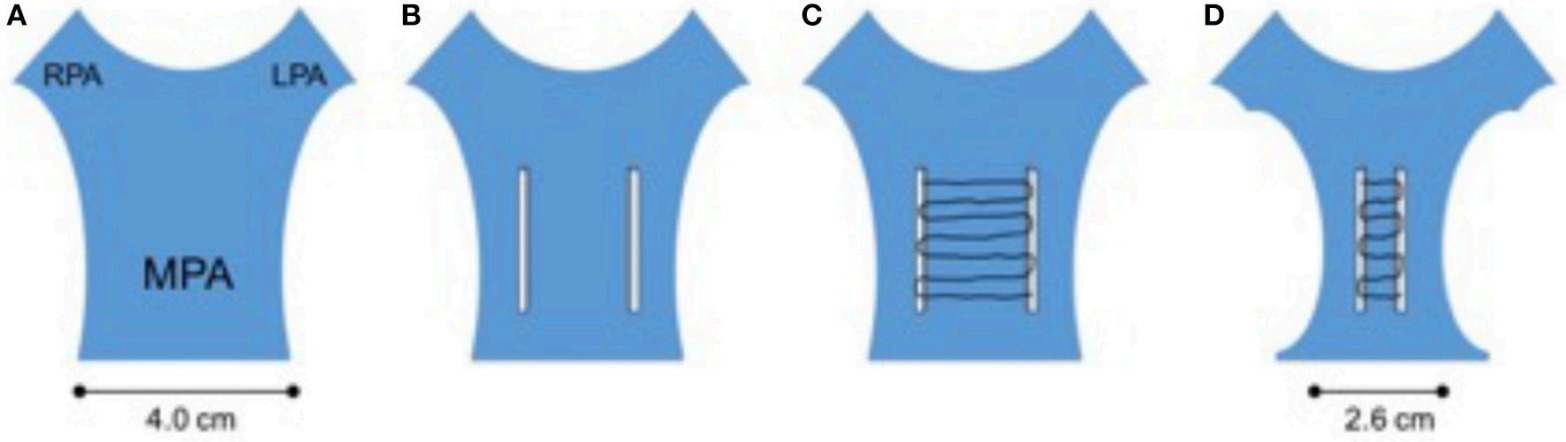
Corset plication of the grossly dilated outflow tract. In this hybrid technique, the surgeon employs 2 widely space teflon strips individually sutured onto the rvot and mpa **(B)**. These strips are then connected with a continuous suture which when pulled causes a corset-like constriction of the outflow **(C)**. This will usually allow safe deployment of currently available percutaneous valves. The initially huge outflow can usually be safely reduced to achieve a diameter 20–30% smaller than the original **(A,D)**.

These strips can then be drawn together like a corset with a continuous suture to decrease the outflow tract size until appropriate. After the outflow tract has been assessed angiographically and by balloon interrogation, the valve can be implanted via the femoral or jugular artery or via a direct puncture in the antero-inferior surface of the right ventricle (Figure 2).

To the interventionist and indeed to most non-invasive cardiologists, this seems like an excellent compromise; avoiding cardiopulmonary bypass, potential circulatory arrest, and all the incumbent comorbidity and pathophysiological insults. From a surgical standpoint however, often the biggest challenge is the redo sternotomy and dissection; once this is achieved, cardiopulmonary bypass is a relatively minor technical challenge. When performing a significant degree of plication, one also needs to be concerned about the potential of tearing the MPA; which could lead to huge blood loss and require the conversion of a potentially elective bypass run into an emergency one. This has been one reason why our team (along with some others) have used the long Teflon pledgets which theoretically decrease the risk of tearing the outflow.

### Use of stents to build landing zones for valve implantation

The use of modern large caliber stents to provide stable scaffolds for valve implantation can be tailored to each individual patient's anatomy. Overall, the strategies can be divided into two types.

The first is simply to pack the outflow tract with coaxial concentric stents which eventually reduce the lumen to a size which can securely grip a percutaneous valve. This can take as many as 5 or 6 stents but can decrease the lumen diameter by >1 mm per stent depending on the stent design. Usually operators have used a combination of stents with high radial strength to provide support, and covered stents providing more bulk in order to achieve a reduction in diameter (13). The other strategy is to utilize the branch pulmonary arteries as anchor points. This can be done either by interlocking stents in a “y”-configuration from both pulmonary branches to the MPA. This creates an area of diameter limited stent support in the outflow tract into which a valve can be implanted. A simpler version of this is to telescope several stents from the LPA into the MPA (14). Determining which technique to adopt is dependent upon the individual anatomical arrangement of the MPA and branches. Although paravalvar leak would seem like an obvious sequela, the behavior of the compliant outflow tract after resolution of any stenosis and reduction in regurgitation may shrink the outflow tract toward the stent, limiting this phenomenon.

### Technology

**Figure 3:**
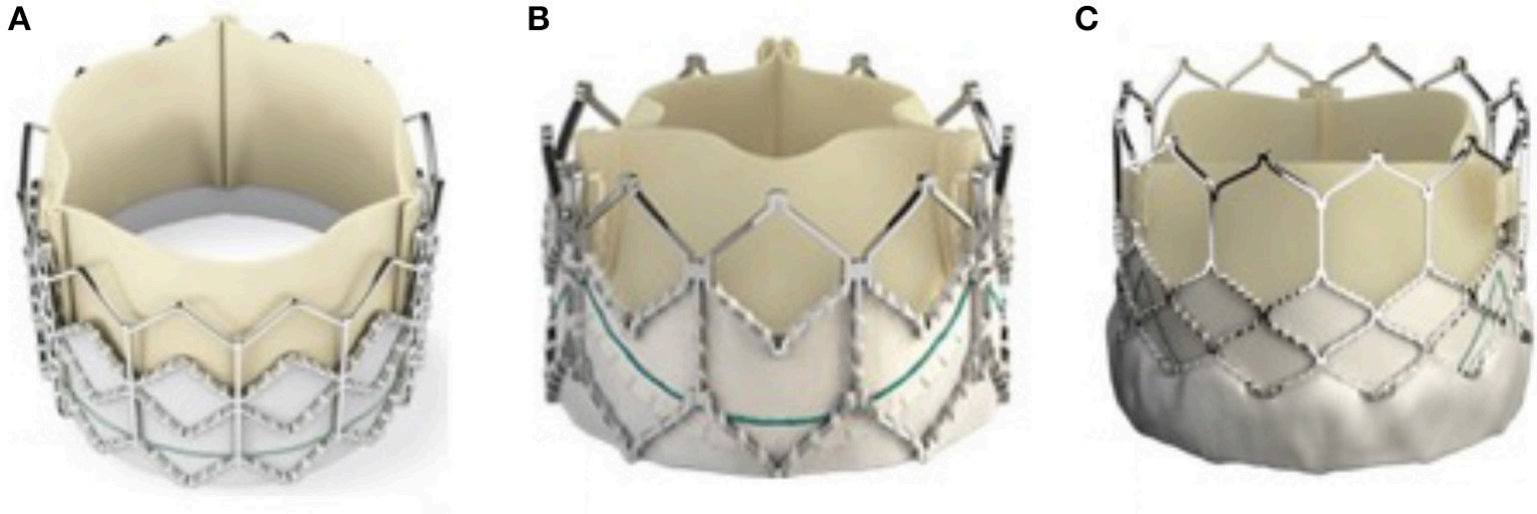
The evolution of the Sapien valve. The original Sapien valve is seen in **(A)** with the XT valve displayed in **(B)** and the latest commercially available iteration the S3 is seen in **(C)**. The changes made to the valve over the years have mostly been to the frame, mainly to combat issues raised during and after deployment in the aortic valve position. The PTFE skirt now allows more expansion of the valve whilst the more open design of the upper portion of the support stent allows easier access to the coronary arteries after deployment.

Since the initial exciting development of the Melody valve, the congenital community has relied on a drip feed of technology from Transcatheter Aortic Valve Replacement (TAVR) to expand the applications for congenital pulmonary patients. This has particularly related to the development of Edwards' Sapien valve series. Each iteration of Sapien valve has progressed based on improvements dictated from the TAVR community. Although the developments have not specifically considered the pulmonic applications, each change has actually improved the applicability for pulmonary deployment (15) (Figure 3). Despite the age of the technology the Melody valve has many comparatively beneficial features compared with even the latest Edwards S3 valve. During delivery through the right atrium and ventricle the Sapien valve is uncovered and may pose a risk to the tricuspid valve; particularly when large amplitude maneuvres need to be made to negotiate the valve into the RVOT. Several cases have been reported of new significant tricuspid valve damage following Sapien valve deployment (16). The Melody valve is encased in a delivery system, known as “Ensemble,” which likely protects the tricuspid valve from being damaged. The nature of the Melody stent frame means that it can expand significantly beyond its advertised diameter; allowing potential space for multiple percutaneous valve in valve procedures. The Sapien stent is very rigid, hence valve in valve procedures will progressively decrease the effective orifice each time a valve is deployed.

**Figure 4:**
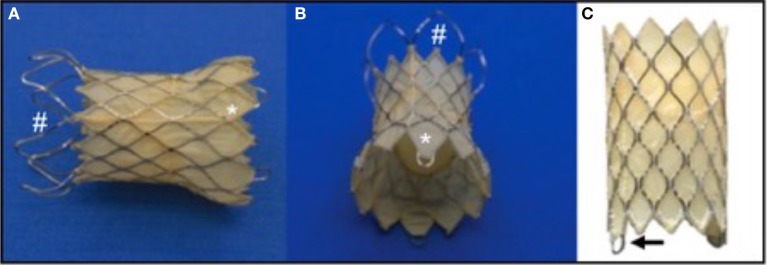
The Medtech Venus P Valve. This self-expanding Nitinol framed valve has a porcine pericardial valve fixed within a covered stent. The covering is also porcine pericardium. The valve comes in two basic designs; the flared design for native outflow tracts **(A,B)**, and the straight design **(C)**, for use in previously stented outflows. **(A,B)**: ^#^represents the uncovered portion of the valve which sits adjacent to the pulmonary bifurcation. *shows the covered portion which sits in the RVOT. The goal is to minimize paravalvar leak whilst decreasing the risk of trapping one of the branch pulmonary arteries during deployment. **(C)**: the arrow points to the eyelet which connects the valve to the delivery system and aids compression of the valve into the delivery system during preparation.

Over the last several years however we have seen novel and specific product development from Medtronic, Venus Medtech, and now Edwards life sciences, related to pulmonary specific equipment. Venus Medtech's Venus P valve has had hundreds of pulmonary implants worldwide and is currently being assessed in a trial in Europe with the intention of securing CE marking (17, 18). It employs a self-expanding dumbbell shaped nitinol frame, partially covered by porcine pericardium and housing a porcine pericardial valve. It has the capacity to deal with outflow tracts up to 34 mm in diameter and has produced good results so far (Figure 4).

**Figure 5:**
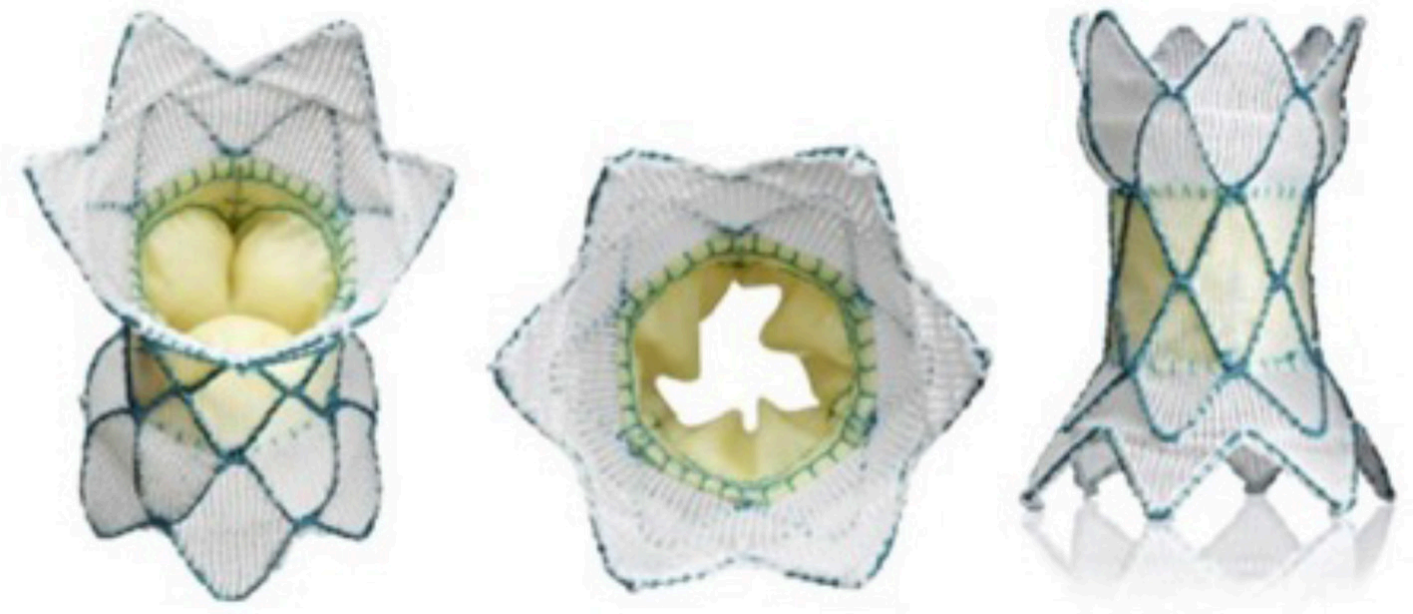
The medtronic harmony valve. The valve is made from porcine pericardial tissue, mounted on a self-expanding nitinol frame. The valved region of the device has an outer diameter of 23.5 mm and is ~55 mm in length. The inflow area is larger than the outflow and both ends are covered unlike the Venus P valve (Figure 3) in which the outflow struts are not covered.

Medtronic have been working on a self-expanding valve frame for many years and are now in the process of clinical trials in North America with the Harmony valve. It has a similar shape to the Venus *P*-valve and also has a Nitinol frame within which is mounted a pericardial valve. The target should be to treat as much as 80% of the population with outflow tract dysfunction with the Harmony valve (Figure 5) (19).

**Figure 6:**
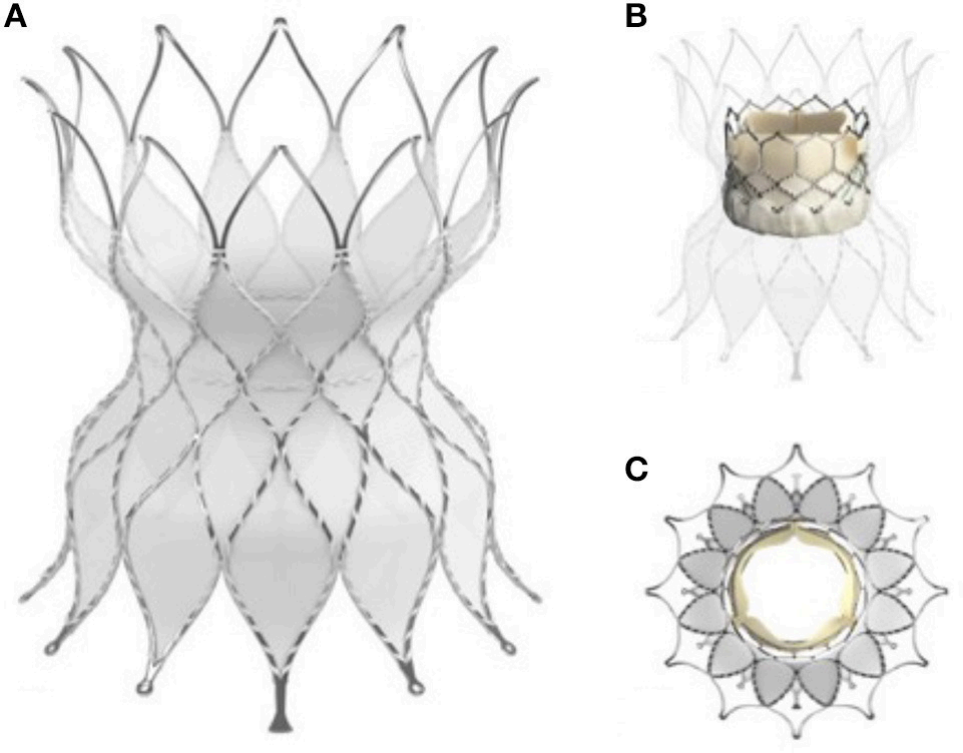
The Edwards Alterra adaptive pre-stent. **(A)** Show the stent, a Nitinol frame almost completely covered excluding the distal end which is uncovered like the Venus P valve (Figure 4) to protect from haemodynamically jailing the branch pulmonary arteries. **(B)** In long axis and **(C)** in short axis show the Alterra device after deployment of the 29 mm Sapien S3 valve inside. The concept of a pre-stent with subsequent valve deployment is a departure from other commercially available and developmental systems.

Whilst Medtronic and Venus have gone with a one piece, stent and valve, Edwards are in the process of clinical trials to evaluate a docking system within which to deploy their current range of valves. The Alterra system comprises a Nitinol framed self-expanding stent which, after delivery in to the RVOT, produces a docking point of fixed size into which a balloon expandable valve can be delivered (Figure 6) (20).

Alongside these innovative delivery platforms, exists the same tissues engineering technology that is being developed for surgical valve platforms. If one can mount a stem cell tissue engineered valve surgically then why not mount it on a percutaneously deliverable platform? (7, 8).

Assuming that these technologies mature into widely available commercial devices, only the most extreme anatomies will not be amenable to percutaneous treatment, and hybrid techniques may be an option in these cases.

## Discussion

Despite the fact that surgical pulmonary valve replacement is a relatively technically simple operation, if we can show non-inferiority using percutaneous valve implantation then this should be the procedure of choice. The recovery time, morbidity and psychological impact on the patient are clearly in favor of developing percutaneous techniques. As part of our decision making process, we must always need to consider the patient's point of view; if there is a technique which may avoid cardiopulmonary bypass, should this not be offered as a first choice? (21).

There is currently huge discussion and debate about the incidence of endocarditis and how it varies dependant on the valve implanted, the technique used and the substrate into which the valve is implanted. The diagnosis of prosthetic valve related endocarditis is poorly standardized within units as well as between units. In some cases, a positive blood culture in a clinically well patient can lead to 6 weeks of intravenous antibiotic treatment and firm diagnosis, whereas in other situations absence of surgical re-intervention can negate a firm clinical and laboratory diagnosis previously made. The fact that manuscripts still appear regularly with new guidelines and criteria for endocarditis should caution us particularly when reviewing large retrospective cohort studies (22, 23). There is slowly growing evidence that the bovine jugular graft, used in the surgically placed Contegra and percutaneously placed Melody valve (both Medtronic Inc., Minneapolis, MN) have an intrinsic proclivity to infection (24, 25). Despite this, the currently commercially available valves mean that for many patients there are no good alternatives surgically or interventionally to the bovine jugular vein. At present, although theories abound, there is an absence of definitive data to trump partisan viewpoints expressed by those, too often with academic and financial ties to industry. The endocarditis story will continue to be a hot topic whilst the relative number of cases and implanted valves is small (26–28).

Are the valves we place percutaneously as durable as surgically placed bioprosthetic valves? We do not know. Not just because we lack good, long-term data for percutaneous valves. There is also a paucity of high quality follow-up for performance of surgically placed valves in the pulmonary position. If we want to get high quality scrutable data we must move away from only looking at “freedom from death or re-intervention.” More subtle and telling parameters such as signs of early functional degeneration of prosthetic valves need to be considered. It is true that the most crucial marker of success for a patient relates to the time period between interventions, but as scientists we need to look for early evidence of subclinical valve dysfunction.

Percutaneous valve placement is not without potential complications, particularly as we push the envelope toward more extreme anatomic and physiologic scenarios. Aside from the chronic specter of endocarditis, the main concerns relate to acute procedurally related complications. Good case planning and multidisciplinary communication should allow for appropriate stabilization and, if necessary surgical intervention in serious situations.

Valve Embolization: In cases where the landing zone for the prosthetic valve is large and compliant, there is an appreciable risk of the valve migrating or embolizing during the implantation process. The most likely time for this is immediately after deployment of the valve in the outflow tract. In such a case, the guide wire over which the valve has been deployed should still be in position; limiting the movement of the valve along the course of the wire. This may still result in ventricular ectopy and decreased cardiac output due to arrhythmia, partial outflow or inflow obstruction and regurgitation of the tricuspid valve. Usually, despite embolization the patients hemodynamics will remain stable allowing a considered approach to remedy the situation. The options include attempting to recapture the valve using a balloon and redeploying it or opening the patient's chest to facilitate hybrid stabilization of the valve without going “on pump” or urgently moving to cardiopulmonary bypass and surgical removal of the embolised valve with subsequent surgical valve replacement (29, 30).

RV-PA Conduit rupture: When significantly increasing the size of a patients outflow to facilitate implantation of an appropriately sized valve, a stiff, calcified or sclerotic outflow tract might respond by splitting instead of stretching in the face of a high pressure balloon expansion. This is well described in literature. Despite the dramatic nature of the description of this complication there are very few reports of acute severe haemodynamic compromise. Patients at risk of outflow tract rupture will have had previous (often several) open heart procedures leading to dense scarring and adhesions replacing the pericardial space; this usually results in rapid and complete or partial containment of bleeding from the outflow tract. In such a case, the extent of the tear and the condition of the patient should determine if the appropriate action is to seal the tear with covered stent implantation or to move toward formal surgical exploration and repair (31).

Significant tricuspid valve regurgitation: Damage to the tricuspid valve is seen rarely. It is probably due to aggressive manipulation of the valve delivery system to achieve deployment position in the outflow tract. It could possibly be a phenomenon mainly related to the current generation of Sapien valve deployment systems, which necessitate maneuvring the uncovered crimped valve through the tricuspid valve apparatus (16).

**Figure 7:**
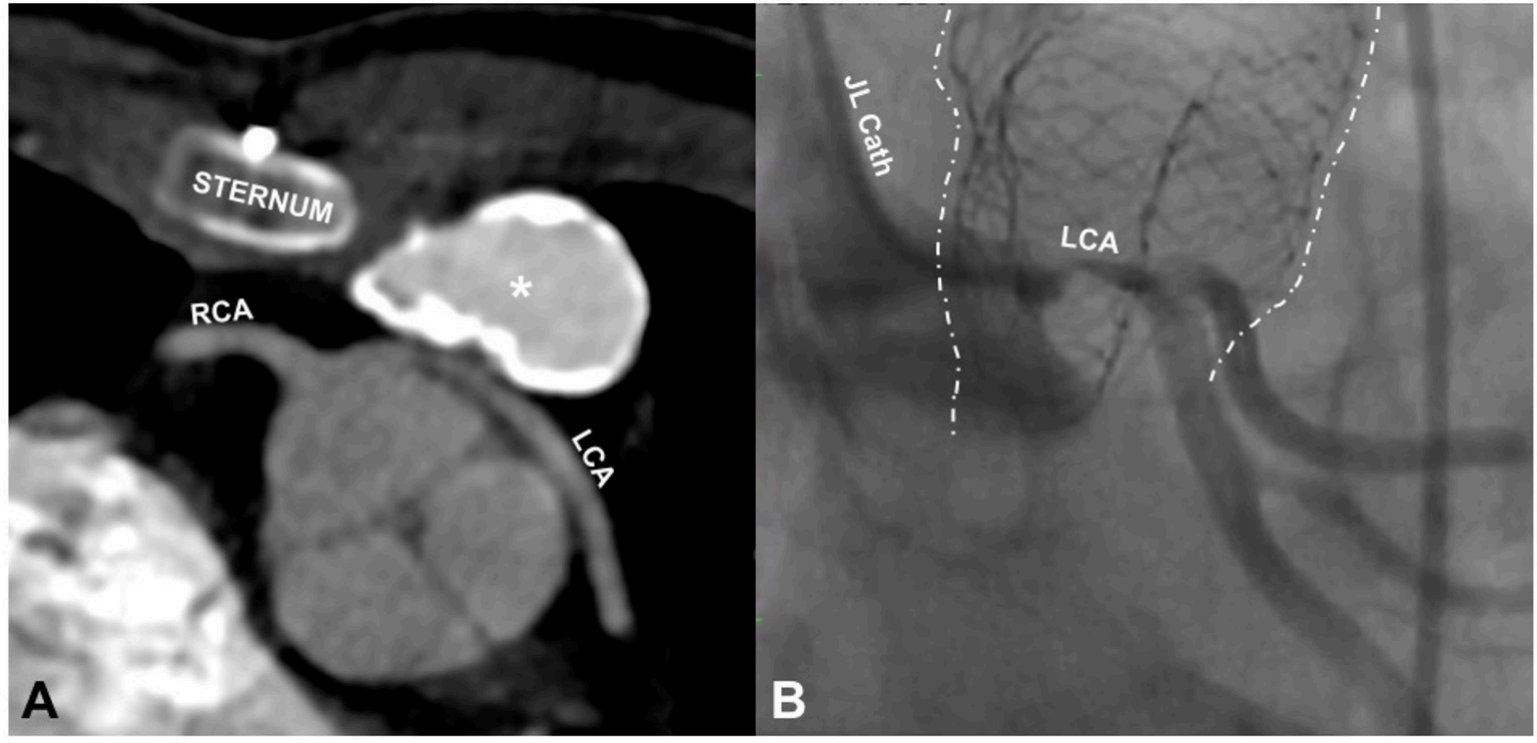
Coronary compression. **(A)** is an axial slice from the CT scan of a 14 year old male showing compression of the left main stem coronary artery (LCA) by a 20 mm Contegra conduit (*), placed 6 years before the CT scan. The origin of this coronary is abnormal, arising from the right coronary sinus beside the right coronary (RCA) before wrapping around the aortic route. The coronary anomaly and the important distortion of the coronary were unrecognized until the patient presented with ventricular tachycardia during a basketball game. **(B)** shows a left coronary angiogram in a different patient (30 year old male) taken during cannulation for ECMO following compression of the left coronary by an outflow tract stent (dotted white lines) placed in preparation for a pulmonary valve implant. The stent is distorted, having been partially crushed by chest compressions after haemodynamic collapse. The coronary label (LCA) is placed directly above the area of near total occlusion. The patient had a surgical valve replacement and was discharged home 5 days later.

Coronary artery compression (Figure 7): The ominous possibility of compression of a coronary artery by the expansion of the valve during deployment can be catastrophic, resulting in acute ischaemic ventricular dysfunction with a drop in cardiac output. Practice development has taken this complication into account and extreme scrutiny and geographical challenges of the relationship of the coronary arteries to the outflow tract are quite consistent among experienced operators (32, 33).

Overall the reported incidence of life-threatening events is very low; however given the excellent safety profile of surgical valve replacement, we must strive to maintain minimal mortality and morbidity as we expand the indications for this procedure.

The development of percutaneous technology in this area is so rapid that that the future place of routine surgical valve replacement is uncertain. The ideal valve should be one which will last the entire life of the patient and can be implanted with minimal risk and minimal disruption to the patient; suggesting that a percutaneously deliverable stem-cell tissue engineered valve is the ultimate goal. We hope that collaboration with surgeons will develop to allow hybrid cases in more challenging anatomies to be routine. With such a collaborative approach, the near future should see pulmonary valve replacement becoming at most a minimally invasive operation and at best a purely percutaneous exercise.

## Author contributions

The author confirms being the sole contributor of this work and approved it for publication.

### Conflict of interest statement

The author declares that the research was conducted in the absence of any commercial or financial relationships that could be construed as a potential conflict of interest.
